# Exploring the factors associated with health literacy among adolescents in Hong Kong

**DOI:** 10.1038/s41598-025-28900-y

**Published:** 2026-01-30

**Authors:** Claire Chenwen Zhong, Mingtao Chen, Zehuan Yang, Zhaojun Li, Vera M. W. Keung, Amelia Lo, Calvin Cheung, Junjie Huang, Martin C. S. Wong

**Affiliations:** 1 The Jockey Club School of Public Health and Primary Care, Faculty of Medicine, The Chinese University of Hong Kong, Shatin, 999077 Hong Kong SAR China; 2 Centre for Health Education and Health Promotion, Faculty of Medicine, The Chinese University of Hong Kong, Shatin, Hong Kong SAR China

**Keywords:** Public Health, Adolescents, Health Literacy, Psychosocial factors, Hong Kong, Health care, Psychology, Psychology, Risk factors

## Abstract

Health literacy is crucial for promoting healthy behaviors and is linked to improved health outcomes among adolescents, yet global levels remain suboptimal. In Hong Kong, where rising rates of obesity and related health issues among adolescents are of increasing concern, understanding health literacy in this population is essential. However, current interventions of health literacy face challenges in effectively addressing the differences between the target groups. Therefore, this study aims to investigate factors associated with health literacy among adolescents in Hong Kong, to provide evidence for identifying groups for intervention implementation. This is a cross-sectional study conducted in Hong Kong. Secondary school students from grades 1 to 4 in Hong Kong were recruited to complete a 98-item survey measuring socio-demographic information, socioeconomic status (SES), health behaviors, mental toughness, and health literacy. Group differences were analyzed using t-tests and ANOVA. Univariable and multivariable linear regression models examined factors associated with adolescent health literacy. A total of 1423 adolescents were included in this study. The mean health literacy score was 57.24 (SD = 17.04) in the adolescents. Multivariable regression showed that aged over 14 years (β = 3.79), medium SES (β = 2.91), high SES (β = 4.80), adequate vegetable intake (β = 0.041), adequate fruit intake (β = 3.13), and mental toughness (β = 10.69) were positively related to overall health literacy. Smoking (β = − 7.38), alcohol drinking (β = − 4.03), excessive electronic game screen time (β = − 2.06), and male gender (β = − 3.86) were negatively associated with overall health literacy. Mental toughness (β = 4.27–12.43) showed a strong positive association across all health literacy sub-domains. This study identified high-risk groups with regard to health literacy among adolescents, including younger individuals, males, those with lower socioeconomic status, poor dietary habits, lower levels of mental toughness, and those engaging in risky behaviors. Future policies and interventions should specifically target these groups to optimize the effectiveness of subsequent initiatives.

**Supplementary Information:**

The online version contains supplementary material available at 10.1038/s41598-025-28900-y.

## Introduction

Health literacy among adolescents has become an increasingly important public health concern. As a key determinant of health behavior, health literacy refers to an individual’s ability to access, understand, evaluate, and apply health-related information to make informed decisions^1–3^. During adolescence—a critical developmental stage—health literacy plays a particularly vital role in shaping long-term health outcomes^4,5^. Previous research has shown that health literacy is a potential influence on adolescent health behavior^6^. Adolescents with low health literacy are more likely to engage in risky behaviors, such as smoking, alcohol consumption, and substance use^6–10^. Likewise, adolescents with a low level of health literacy are more likely to exhibit adverse mental health symptoms^11^. However, studies have consistently shown that the level of health literacy among adolescents is generally low worldwide. Approximately 59% of adolescents in eight European countries were found to have limited health literacy^12^; 38% of adolescents in China had a low level of health literacy^13^, while over 30% of adolescents in Taiwan had inadequate or problematic health literacy^14^.

In Hong Kong, studies on health literacy are limited. An earlier study preliminarily indicated that approximately 75% adolescents in Hong Kong exhibited a limited health literacy level^15^. Similarly, local surveys indicate that the health status of adolescents in Hong Kong is concerning, with more than 20% of secondary school students classified as overweight and approximately 60% experiencing vision problems during the 2022/2023 academic year, which demonstrates a more severe situation compared to schoolchildren in the early stages^16^. These phenomena underscore an urgent need to strengthen health literacy in adolescents.

In response, a growing number of interventions have been implemented in recent years to promote health literacy in adolescents^17–19^. However, most of these interventions have focused on improving specific health outcomes rather than health literacy itself^19,20^. Moreover, emerging research indicates that the effectiveness of such interventions may differ across subgroups due to variations in demographic and contextual factors such as age, gender, socioeconomic status, and educational background^17,21^. As a result, there is increasing interest in identifying the factors associated with adolescent health literacy to better tailor and optimize intervention strategies.

The integrated conceptual model of health literacy proposed by Sørensen and colleagues emphasizes that the potential antecedents of health literacy encompass not only social and environmental determinants but also individual factors such as age, gender, behavior, and personal beliefs, which are closely associated with adolescents’ health literacy competencies^22^. To date, studies exploring the determinants of adolescent health literacy have mainly focused on sociodemographic variables^23,24^. While this line of inquiry has provided important insights, it remains limited in scope. Recent evidence suggests that health-related behaviors—such as physical activity, diet, and sleep patterns—as well as psychosocial characteristics, including self-efficacy and mental toughness, may play crucial roles in shaping health literacy levels. Studies have shown that adolescents who engage in regular physical activity report higher health literacy and more robust health-promoting lifestyles; for instance, Taiwanese youth exercising three or more times per week exhibited significantly higher health literacy, healthier lifestyle profiles, and better emotional stability^25^. In terms of psychosocial factors, self-efficacy has been recognized as a determinant of adolescents’ health behaviors. Higher self-efficacy correlates with improved stress management and healthier lifestyle choices, likely mediated by better health information engagement^26^.

Nevertheless, current research frequently investigates these factors in isolation and lacks a comprehensive framework for understanding their interactions and effects on adolescent health literacy. Specifically, in Hong Kong, the scarcity of pertinent studies may present obstacles to implementing effective health literacy interventions. Therefore, this study aims to explore the association of factors across various domains at the individual level among adolescents—including demographic characteristics, health behaviors, and psychological attributes—with adolescent health literacy, and to identify high-risk subgroups, thereby provide evidence-based recommendations for the development of more targeted and effective health literacy interventions. Specifically, the research question guiding the study was: “What are the individual-level factors related to health literacy among adolescents in Hong Kong?”.

## Methods

### Study design and participants

This was a large population-based study targeting adolescents in Hong Kong. Eligible participants were secondary school students from Grade 1 to Grade 4 (equivalent to Secondary 1 to 4 in the local education system). In this study, a cluster sampling approach was employed, and a total of 20 schools participating in the “Health Education Built-on Project,” conducted by the Centre for Health Education and Health Promotion at the Chinese University of Hong Kong, were invited and consented to participate. Upon agreeing to participate, each school was asked to select one grade level (from Grade 1 to Grade 4) to take part in the study. We estimated that a minimum of 1068 participants is required under the assumption of 50% of participants report outcome of interest (proportion p) with 5% type I error and achieve a precision level of 0.03 based on the following formula: [N = 1.96 × p(1-p)/ precision^2^]^27^. With the schools’ consent, hard copies of the questionnaires were distributed to all participating schools. Students completed the questionnaire during class time, which took approximately 20 to 30 min. Trained teachers administered the survey using standardized instructions and were available to answer any questions raised by student during the process.

### Data collection

This study was approved by the Survey and Behavioural Research Ethics Committee of The Chinese University of Hong Kong (SBRE-24-0029) prior to initiation. The study design and reporting were performed in accordance with the Declaration of Helsinki. Informed consent was collected from all students who participated in the study, as well as from their parents, before they filled out the questionnaire. Students participating in the study have been informed of the voluntary nature of the study, the confidential and anonymous nature of the study, and the right to withdraw from the study at any time. All completed questionnaires are kept in sealed envelopes upon completion and returned to the research office without being accessed by the teacher or principal. To further ensure confidentiality, participants’ personal information has been anonymized and will not be associated with the data in any way.

### Survey instrument

Data were collected using a structured self-administered questionnaire comprising five major domains: socio-demographic characteristics, socio-economic status (SES), health behaviors, mental toughness, and health literacy. The scales employed in this study were translated into Chinese through a forward–backward translation process conducted by the research team to ensure accuracy. Visual material and plain language were used to construct the questionnaire, enhancing its readability and comprehension.

Adolescents’ health literacy was assessed using the validated Health Literacy Measure for Adolescents (HELMA), which consists of 44 items across eight dimensions: access to health information, reading ability, comprehension, appraisal, use of information, communication, self-efficacy, and numeracy^28^. Each item was rated on a 5-point Likert scale, with higher scores indicating better health literacy. The scores for the overall scale and its subscales range from 0 to 100. The Cronbach’s alpha coefficient for this scale is 0.96 in this study.

Socio-economic status was measured using four items from the Family Affluence Scale II: ownership of a private bedroom, number of household cars, number of household computers, and frequency of travel abroad in the past 12 months. A composite score was calculated and used to classify SES into three categories: low (0–3), medium (4–5), and high (6–7)^29^. The Cronbach’s alpha coefficient for this scale is 0.34 in this study.

The measurement of health behaviors was adapted from the Global School-Based Student Health Survey (GSHS) developed by the World Health Organization (WHO)^30^, which has been widely adopted in previous studies^31,32^, include domains in dietary habits (e.g., daily breakfast consumption, vegetable and fruit intake), physical activity, screen time on video, electronic games and social media, sufficient sleep, smoking, and alcohol consumption in the past 30 days.

Of these, participants who reported consuming fewer than three servings of vegetables and fewer than two servings of fruit daily in the past seven days were classified as having insufficient vegetable and fruit intake, respectively, in accordance with the guidelines established by the Hong Kong Centre for Health Protection^33^. Participants who reported engaging in at least an average of 60 min per day of moderate-to-vigorous activity within the past seven days were classified as sufficient physical activity as recommended by the WHO^34^. Sufficient sleep refers to at least 8 h of sleep duration per day, as recommended by international health guidelines^35^. Participants who reported ≥ 2 h of screen exposure on video content, electronic gaming, or social media per day on weekdays, on average, were categorized as having excessive screen time exposure, as recommended by existing guidelines from Canada^36^. In addition, participants were also required to report their perceived body weight status, with self-perceived overweight classified as “slightly overweight” or “very overweight” based solely on self-report.

Mental toughness was measured using the Mental Toughness Scale for Adolescents (MTS-A), assessing six dimensions: challenge (seeking opportunities for self-development), commitment (belief in and ability to achieve goals), emotional control (ability to regulate emotions), life control (beliefs in shaping one’s own life), interpersonal confidence (confidence in social situation), and confidence in ability (confidence in facing difficulties). Each dimension includes three items scored on a 4-point Likert scale, with higher scores indicating greater mental toughness^37^. The MTS-A scale demonstrated satisfactory performance with Cronbach’s alpha coefficient of 0.87.

### Data analysis

The images of the questionnaire were stored in a computer, and the responses were identified and entered using Remark Office OMR 11 (Gravic Inc., Malvern, PA, USA). A member of the research team was responsible for quality control and performed cross-checks to ensure the accuracy of data entry. Descriptive statistics were calculated for all variables. Difference in HELMA scores across groups were examined using t-tests and one-way analysis of variance (ANOVA). To identify factors independently associated with health literacy, univariable and multivariable linear regression analyses were performed. The linear regression model included 15 explanatory variables, such as age, sex, SES, smoking, alcohol consumption, dietary and physical activity behaviors, sufficient sleep, excessive screen time, perceived obesity, and MTS-A. Beta coefficients (βs) and their 95% confidence intervals (CIs) were reported. Missing data were addressed through a complete case analysis approach, whereby questionnaires with missing values were excluded from the analysis. The Variance Inflation Factors (VIF) test was utilized to assess multicollinearity among the variables. Variables with a generalized VIF (GVIF) greater than 5 were regarded as indicative of multicollinearity and were consequently omitted from the regression model^38^. Statistical significance was defined as *p* < 0.05. All analyses were performed using R-4.2.1.

## Results

### Socio-demographic characteristics

**Fig. 1:**
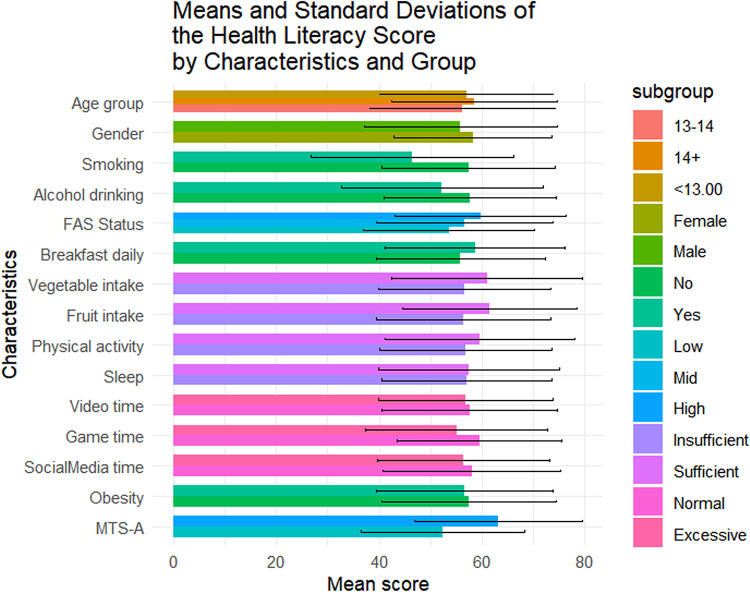
Means and standard deviations of the health literacy measure for adolescents by characteristics and group.

**Fig. 2:**
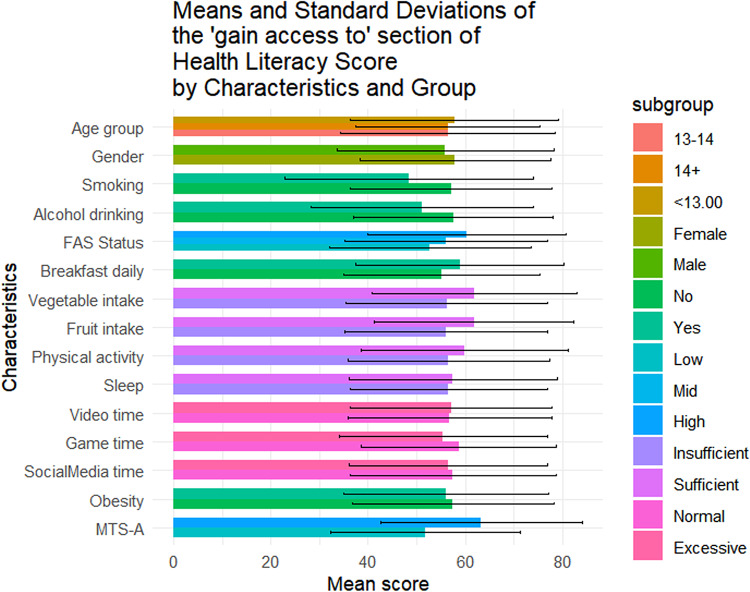
Means and standard deviations of the “Gain access to” section by characteristics and group.

**Fig. 3:**
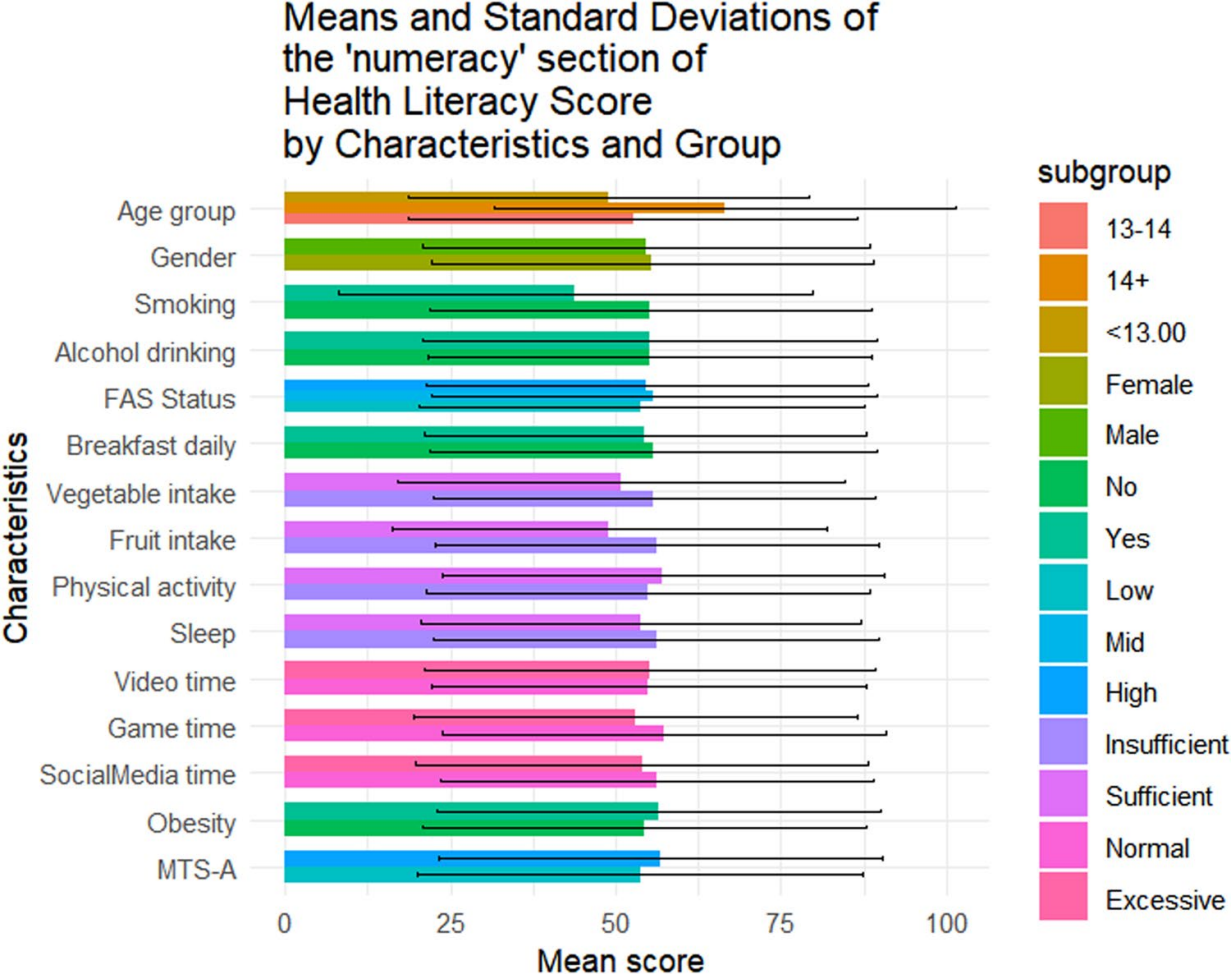
Means and standard deviations of the “Numeracy” section by characteristics and group.

**Fig. 4:**
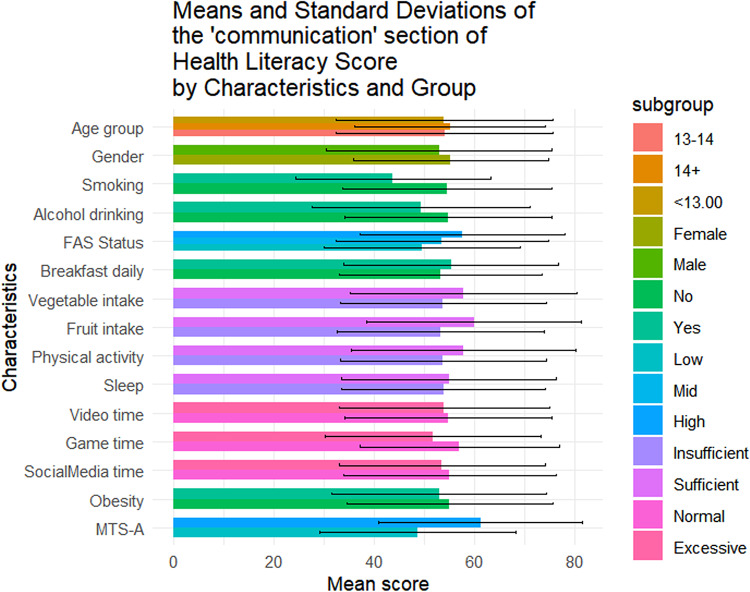
Means and standard deviations of the “Communication” section by characteristics and group.

**Fig. 5:**
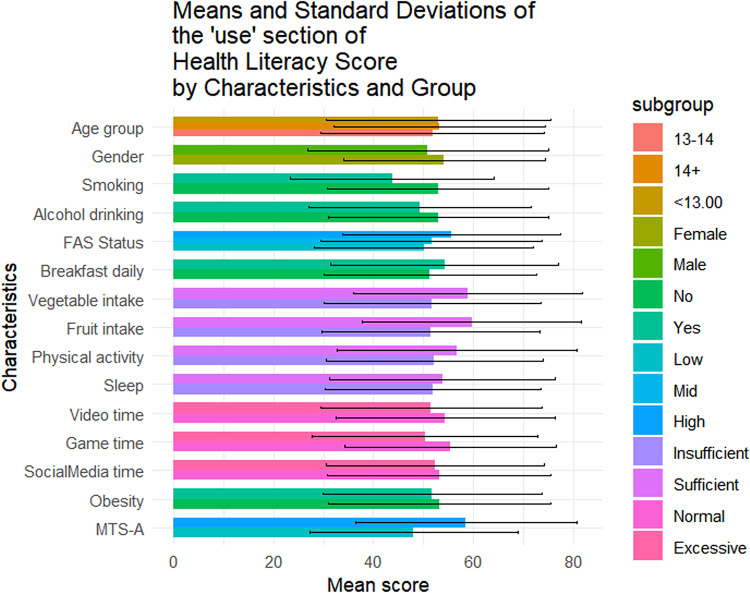
Means and standard deviations of the “Use” section by characteristics and group.

**Fig. 6:**
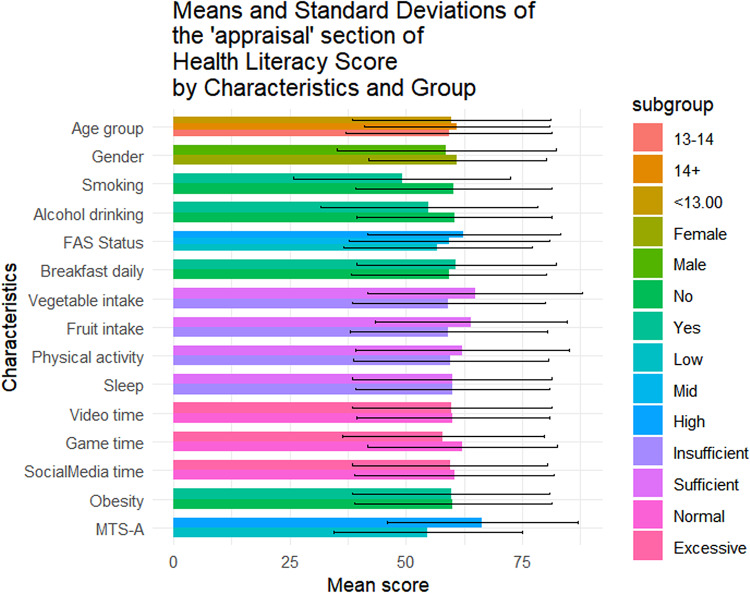
Means and standard deviations of the “Appraisal” section by characteristics and group.

**Fig. 7:**
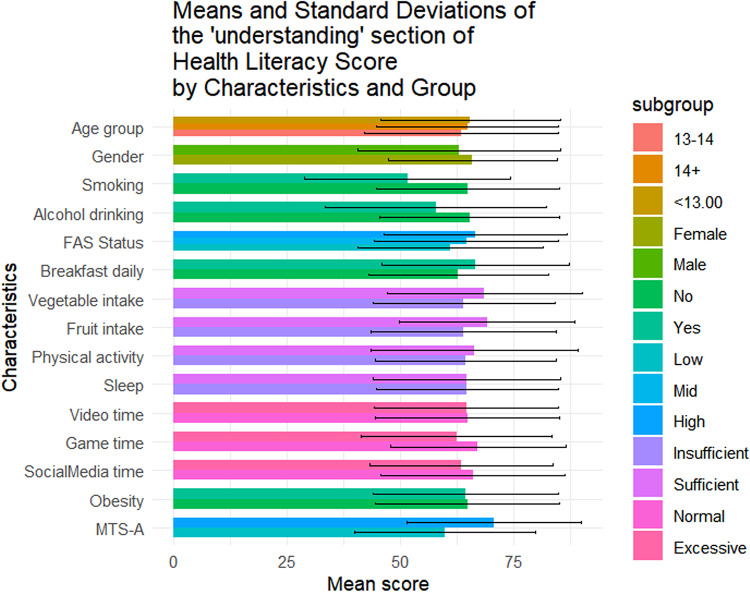
Means and standard deviations of the “Understanding” section by characteristics and group.

**Fig. 8:**
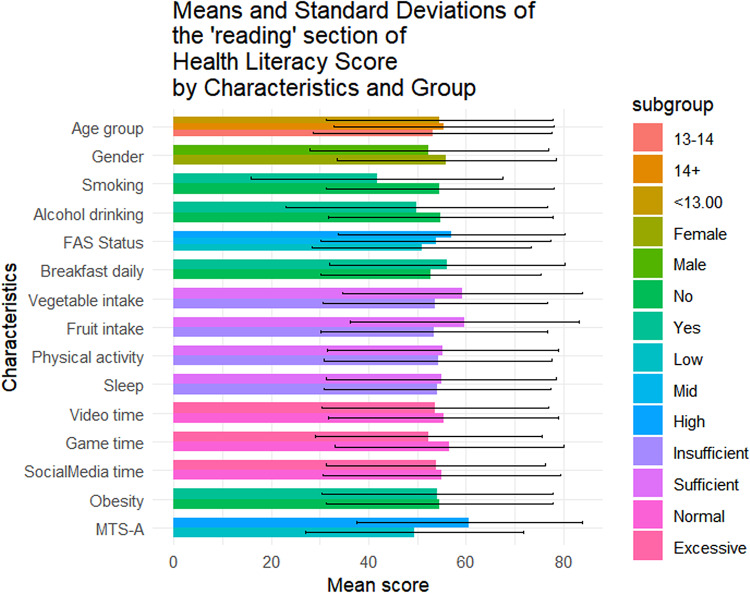
Means and standard deviations of the “Reading” section by characteristics and group.

**Fig. 9:**
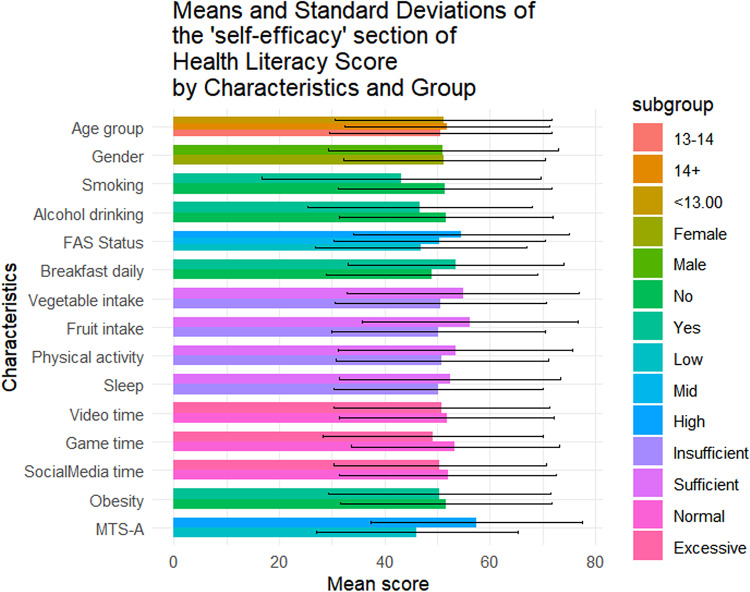
Means and standard deviations of the “Self-efficacy” section by characteristics and group.

Table 1 summarizes the sociodemographic characteristics of the 1423 adolescents included in this study. Of them, 56.4% (n = 802) were female, 42.4% (n = 604) were aged below 13, and 49.0% (n = 697) came from families with medium SES. The mean overall health literacy score was 57.24 (SD = 17.04), with subdimension scoring from 51.21(20.38) to 64.62(20.33). Results of t-tests and ANOVA revealed that higher health literacy scores were observed among females (*p* = 0.011), adolescents from higher SES backgrounds(*p* < 0.001), non-smokers(*p* = 0.014), those with no history of alcohol use(*p* = 0.004), those who regularly ate breakfast(*p* = 0.002), consumed adequate fruits and vegetables(*p* < 0.05), spent not excessive time on electronic games(*p* < 0.001), and reported higher levels of mental toughness(*p* < 0.001) (Fig. 1). Similar patterns were observed across all health literacy subscales (Figs. 2, 3, 4, 5, 6, 7, 8, 9).

**Table 1:** Participant characteristics and the Health Literacy Measure for Adolescents among the participants.

Variable	Number (%)	Health literacy (mean (sd))	Gain access to	Numeracy	Communication	Use	Appraisal	Understanding	Reading	Self-efficacy
	1423	57.24 (17.04)	56.99 (20.87)	55.09 (33.56)	54.28 (20.86)	52.77 (22.09)	59.99 (21.21)	64.62 (20.33)	54.42 (23.43)	51.21 (20.38)
*Age group*										
< 13 years	604 (42.4%)	57.02 (16.92)	57.75 (21.34)	48.88 (30.28)	53.90 (21.61)	53 (22.55)	59.78 (21.41)	65.39 (19.80)	54.58 (23.27)	51.17 (20.54)
13–14 years	405 (28.5%)	56.30 (18.06)	56.40 (22.02)	52.63 (33.92)	54 (21.55)	51.84 (22.32)	59.26 (22.22)	63.35 (21.33)	53.14 (24.51)	50.63 (21.02)
> 14 years	414 (29.1%)	58.48 (16.12)	56.46 (18.94)	66.57 (34.90)	55.11 (19.02)	53.34 (21.18)	60.99 (19.88)	64.73 (20.08)	55.43 (22.55)	51.83 (19.51)
*P value*		0.172	0.499	** < 0.001**	0.627	0.588	0.483	0.291	0.364	0.703
*Sex*										
Female	802 (56.4%)	58.28 (15.40)	57.86 (19.64)	55.53 (33.35)	55.29 (19.43)	54.20 (20.28)	61.01 (19.08)	65.90 (18.55)	56 (22.48)	51.31 (19.18)
Male	621 (43.6%)	55.90 (18.88)	55.87 (22.32)	54.54 (33.83)	52.97 (22.53)	50.92 (24.11)	58.66 (23.62)	62.96 (22.33)	52.37 (24.46)	51.08 (21.84)
*P value*		**0.011**	0.080	0.581	**0.041**	**0.006**	**0.044**	**0.008**	**0.004**	0.834
*SES [0–9]*										
Low [0–2]	236 (16.6%)	53.58 (16.66)	52.78 (20.69)	53.88 (33.66)	49.50 (19.62)	50.13 (21.94)	56.82 (20.26)	60.95 (20.43)	50.91 (22.47)	46.85 (20.10)
Mid [3–5]	697 (49.0%)	56.71 (17.19)	56.05 (20.95)	55.81 (33.64)	53.55 (21.18)	51.65 (22.16)	59.35 (21.66)	64.55 (20.30)	53.78 (23.63)	50.34 (20.01)
High [6–9]	490 (34.4%)	59.76 (16.66)	60.35 (20.36)	54.66 (33.43)	57.62 (20.47)	55.62 (21.80)	62.42 (20.78)	66.47 (20.13)	57.01 (23.35)	54.54 (20.54)
*P value*		** < 0.001**	** < 0.001**	0.703	** < 0.001**	**0.001**	**0.002**	**0.003**	**0.003**	** < 0.001**
*Smoking*										
No	1400 (98.4%)	57.42 (16.94)	57.13 (20.76)	55.28 (33.50)	54.45 (20.85)	52.92 (22.09)	60.16 (21.13)	64.83 (20.23)	54.62 (23.34)	51.34 (20.25)
Yes	23 (1.6%)	46.39 (19.75)	48.48 (25.60)	43.84 (35.82)	43.75 (19.52)	43.75 (20.38)	49.13 (23.29)	51.52 (22.79)	41.74 (25.88)	43.21 (26.51)
*P value*		**0.014**	0.121	0.142	**0.016**	**0.044**	**0.034**	**0.011**	**0.027**	0.157
*Alcohol drinking*										
No	1304 (91.6%)	57.69 (16.71)	57.53 (20.59)	55.10 (33.49)	54.73 (20.73)	53.08 (22.06)	60.44 (20.96)	65.24 (19.82)	54.84 (23.05)	51.62 (20.25)
Yes	119 (8.4%)	52.29 (19.67)	51.05 (22.91)	55.04 (34.36)	49.29 (21.77)	49.32 (22.21)	54.96 (23.26)	57.84 (24.38)	49.79 (26.88)	46.64 (21.26)
*P value*		**0.004**	**0.003**	0.986	**0.010**	0.079	**0.014**	**0.002**	**0.049**	**0.015**
*Having breakfast 7 days in a week*										
No	726 (51%)	55.86 (16.46)	55.10 (20.14)	55.72 (33.73)	53.29 (20.22)	51.36 (21.31)	59.26 (20.94)	62.76 (19.91)	52.78 (22.54)	48.96 (20.01)
Yes	697 (49%)	58.67 (17.52)	58.95 (21.43)	54.45 (33.39)	55.31 (21.48)	54.23 (22.79)	60.75 (21.47)	66.55 (20.60)	56.12 (24.21)	53.55 (20.51)
*P value*		**0.002**	**0.001**	0.476	0.068	**0.014**	0.186	** < 0.001**	**0.007**	** < 0.001**
*Sufficient vegetable intake*										
No	1225 (86.1%)	56.63 (16.72)	56.20 (20.72)	55.78 (33.47)	53.71 (20.53)	51.78 (21.79)	59.20 (20.78)	63.99 (20.06)	53.64 (23.14)	50.62 (20.03)
Yes	198 (13.9%)	61.02 (18.51)	61.87 (21.17)	50.88 (33.88)	57.80 (22.57)	58.90 (22.98)	64.85 (23.13)	68.52 (21.58)	59.22 (24.67)	54.86 (22.10)
*P value*		**0.002**	**0.001**	0.060	**0.017**	** < 0.001**	**0.001**	**0.006**	**0.003**	**0.012**
*Sufficient fruit intake*										
No	1193 (83.8%)	56.63 (16.72)	56.06 (20.80)	56.28 (33.57)	53.20 (20.58)	51.43 (21.87)	59.20 (21.24)	63.76 (20.42)	53.39 (23.30)	50.26 (20.23)
Yes	230 (16.2%)	61.02 (18.51)	61.78 (20.59)	48.95 (32.86)	59.89 (21.44)	59.70 (21.96)	64.07 (20.59)	69.05 (19.33)	59.72 (23.41)	56.14 (20.44)
*P value*		** < 0.001**	** < 0.001**	**0.002**	** < 0.001**	** < 0.001**	**0.001**	** < 0.001**	** < 0.001**	** < 0.001**
*Sufficient physical activity*										
No	1239 (87.1%)	56.89 (16.81)	56.57 (20.78)	54.79 (33.58)	53.76 (20.57)	52.17 (21.73)	59.66 (20.92)	64.37 (19.94)	54.29 (23.40)	50.88 (20.09)
Yes	184 (12.9%)	59.63 (18.41)	59.84 (21.26)	57.16 (33.41)	57.76 (22.46)	56.79 (24.05)	62.17 (23.01)	66.28 (22.78)	55.27 (23.68)	53.40 (22.15)
*P value*		0.058	0.052	0.371	**0.024**	**0.014**	0.164	0.283	0.600	0.147
*Sufficient sleep*										
No	792 (55.7%)	56.98 (16.57)	56.58 (20.36)	56.19 (33.73)	53.80 (20.36)	51.90 (21.63)	60.04 (20.98)	64.65 (20.06)	54.02 (23.28)	50.26 (19.84)
Yes	631 (44.3%)	57.56 (17.61)	57.50 (21.49)	53.72 (33.31)	54.88 (21.47)	53.85 (22.62)	59.92 (21.51)	64.58 (20.68)	54.91 (23.63)	52.40 (20.98)
*P value*		0.528	0.409	0.169	0.336	0.100	0.918	0.951	0.477	0.051
*Excessive screen time on video*										
No	595 (41.8%)	57.65 (17.11)	56.83 (20.95)	54.97 (32.92)	54.74 (20.74)	54.43 (21.85)	60.11 (20.80)	64.72 (20.35)	55.39 (23.64)	51.79 (20.31)
Yes	828 (58.2%)	56.94 (16.99)	57.10 (20.82)	55.18 (34.02)	53.94 (20.96)	51.57 (22.19)	59.90 (21.51)	64.54 (20.33)	53.71 (23.26)	50.79 (20.42)
*P value*		0.441	0.810	0.906	0.476	**0.016**	0.852	0.868	0.183	0.364
*Excessive screen time on electronic game*										
No	683 (48%)	59.58 (16.02)	58.66 (20.12)	57.30 (33.44)	57.01 (19.84)	55.44 (21.25)	62.17 (20.43)	67.10 (19.27)	56.61 (23.44)	53.39 (19.67)
Yes	740 (52%)	55.08 (17.67)	55.45 (21.43)	53.06 (33.55)	51.75 (21.47)	50.30 (22.56)	57.97 (21.72)	62.33 (21.02)	52.39 (23.25)	49.20 (20.82)
*P value*		** < 0.001**	**0.004**	**0.017**	** < 0.001**	** < 0.001**	** < 0.001**	** < 0.001**	**0.001**	** < 0.001**
*Excessive screen time on social media*										
No	691 (48.6%)	58.05 (17.33)	57.50 (21.23)	56.27 (32.76)	55.06 (21.28)	53.14 (22.40)	60.48 (21.46)	65.96 (20.37)	55.01 (24.41)	52.00 (20.62)
Yes	732 (51.4%)	56.47 (16.73)	56.50 (20.52)	53.98 (34.27)	53.54 (20.45)	52.42 (21.79)	59.52 (20.97)	63.35 (20.23)	53.85 (22.46)	50.46 (20.13)
*P value*		0.080	0.366	0.198	0.172	0.538	0.396	**0.016**	0.351	0.155
*Self-reported obesity*										
No	943 (66.3%)	57.52 (16.94)	57.44 (20.77)	54.36 (33.57)	54.98 (20.53)	53.25 (22.19)	60.13 (21.19)	64.77 (20.26)	54.57 (23.27)	51.60 (20.01)
Yes	480 (33.7%)	56.70 (17.23)	56.10 (21.04)	56.55 (33.51)	52.90 (21.46)	51.82 (21.88)	59.70 (21.26)	64.31 (20.48)	54.12 (23.76)	50.43 (21.08)
*P value*		0.394	0.256	0.245	0.081	0.248	0.715	0.688	0.739	0.312
*MTS-A*										
Low	784 (55.1%)	63.22 (16.39)	63.33 (20.80)	56.83 (33.48)	61.23 (20.29)	58.57 (22.19)	66.42 (20.59)	70.56 (19.29)	60.61 (23.16)	57.42 (20.09)
High	639 (44.9%)	52.36 (15.98)	51.82 (19.46)	53.68 (33.57)	48.61 (19.58)	48.04 (20.85)	54.74 (20.25)	59.77 (19.88)	49.37 (22.43)	46.14 (19.18)
*P value*		** < 0.001**	** < 0.001**	0.078	** < 0.001**	** < 0.001**	** < 0.001**	** < 0.001**	** < 0.001**	** < 0.001**

SES, Socio-economic status; Sufficient vegetable intake: Had at least 3 servings of vegetables everyday; Sufficient fruit intake: Had at least 2 servings of fruit everyday; Sufficient physical activity: Moderate/Vigorous exercise >  = 1 h everyday; Sufficient sleep: Sleep at least 8 h; Excessive screen time: Spending over 2 h time of screen; Mental Toughness Scale for Adolescents: The scale [18–72] is composed of six dimensions, including challenge [3–12], commitment [3–12], emotion control [3–12], life control [3–12], confidence in abilities [3–12], and interpersonal confidence [3–12], with a higher score indicating higher mental toughness. MTS > 46 was classified into High group. P-values: T-test and ANOVA test in the Health Literacy Measure for Adolescents among the participants.

Significant values are in bold.

### Linear regression analysis between factors and health literacy

The results of the univariable and multivariable linear regression analyses between factors and health literacy are presented in Table 1. No variables exhibited multicollinearity in the VIF test (Supplementary Table 1). Furthermore, the intercorrelation matrix among the subdomains of health literacy was provided in Supplementary Table 2 and Supplementary Fig. 1, demonstrating that the subdomains represent distinct aspects despite their intercorrelations, with correlation coefficients ranging from 0.07 to 0.80.

As shown in Table 2, adolescents aged over 14 years (β = 3.79, 95% CI: 1.75–5.82, *p* < 0.001) demonstrated significantly higher levels of overall health literacy compared with those aged 12 or below. Similarly, higher SES was linked to improved health literacy (β_medium = 2.91, 95% CI: 0.57–5.25, *p* = 0.015; β_high = 4.80, 95% CI: 2.31–7.29, *p* < 0.001), adequate vegetable intake (β = 2.54, 95% CI: 0.11–4.96, *p* = 0.041), adequate fruit intake (β = 3.13, 95% CI: 0.82–5.44, *p* = 0.008) also contributed positively. Mental toughness emerged as a strong determinant, with each unit increase associated with a 10.69 score improvement in health literacy (β = 10.69, 95% CI: 8.96–12.41, *p* < 0.001). In contrast, smoking (β =  − 7.38, 95% CI: − 14.06 to − 0.70, *p* = 0.030), alcohol consumption (β =  − 4.03, 95% CI: − 7.12 to − 0.94, *p* = 0.011), excessive electronic game use (β =  − 2.06, 95% CI: − 3.85 to − 0.27, *p* = 0.024), and being male (β =  − 3.86, 95% CI: − 5.62 to − 2.09, *p* < 0.001) were all independently associated with reduced overall health literacy.

**Table 2:** Factors associated with the Health Literacy Measure for Adolescents among the participants.

Variable	Univariable beta* (95%CI)	p-value	Multivariable beta (95%CI)	p-value
*Age group*				
< 13 years	reference		reference	
13–14 years	− 0.72 (− 2.87, 1.42)	0.509	1.54 (− 0.48, 3.56)	0.136
> 14 years	1.46 (− 0.68, 3.59)	0.180	3.79 (1.75, 5.82)	** < 0.001**
*Sex*				
Female	reference		reference	
Male	− 2.38 (− 4.17, − 0.60)	**0.009**	− 3.86 (− 5.62, − 2.09)	** < 0.001**
*SES [0–9]*				
*Low [0–2]*	reference		reference	
Mid [3–5]	3.13 (0.63, 5.63)	**0.014**	2.91 (0.57, 5.25)	**0.015**
High [6–9]	6.17 (3.54, 8.80)	** < 0.001**	4.80 (2.31, 7.29)	** < 0.001**
*Smoking*				
No	reference		reference	
Yes	− 11.02 (− 18.03, − 4.02)	**0.002**	− 7.38 (− 14.06, − 0.70)	**0.030**
*Alcohol drinking*				
No	reference		reference	
Yes	− 5.40 (− 8.59, − 2.21)	**0.001**	− 4.03 (− 7.12, − 0.94)	**0.011**
*Having breakfast 7 days in a week*				
No	reference		reference	
Yes	2.81 (1.04, 4.57)	**0.002**	0.86 (− 0.85, 2.58)	0.324
*Sufficient vegetable intake*				
No	reference		reference	
Yes	4.39 (1.84, 6.95)	**0.001**	2.54 (0.11, 4.96)	**0.041**
*Sufficient fruit intake*				
No	reference		reference	
Yes	5.13 (2.74, 7.52)	** < 0.001**	3.13 (0.82, 5.44)	**0.008**
*Sufficient physical activity*				
No	reference		reference	
Yes	2.74 (0.10, 5.38)	**0.042**	1.59 (− 0.95, 4.14)	0.220
*Sufficient sleep*				
No	reference		reference	
Yes	0.58 (− 1.21, 2.36)	0.525	− 0.26 (− 1.98, 1.46)	0.767
*Excessive screen time on video*				
No	reference		reference	
Yes	− 0.71 (− 2.50, 1.09)	0.440	0.80 (− 0.98, 2.58)	0.379
*Excessive screen time on electronic game*				
No	reference		reference	
Yes	− 4.50 (− 6.26, − 2.74)	** < 0.001**	− 2.06 (− 3.85, − 0.27)	**0.024**
*Excessive screen time on social media*				
No	reference		reference	
Yes	− 1.58 (− 3.35, 0.19)	0.080	− 0.59 (− 2.40, 1.23)	0.526
*Self-reported obesity*				
No	reference		reference	
Yes	− 0.82 (− 2.69, 1.05)	0.391	− 0.12 (− 1.87, 1.64)	0.898
*MTS-A*				
Low	reference		reference	
High	10.86 (9.17, 12.55)	** < 0.001**	10.69 (8.96, 12.41)	** < 0.001**

*In linear regression, the beta coefficient indicates the influence of an independent variable on the dependent variable. A one-unit increase in the independent variable results in an average change of the beta coefficient in the dependent variable. Positive values indicate a positive correlation, while negative values indicate a negative correlation.

CI, confidence interval; SES, Socio-economic status; Sufficient vegetable intake: Had at least 3 servings of vegetables everyday; Sufficient fruit intake: Had at least 2 servings of fruit everyday; Sufficient physical activity: Moderate/Vigorous exercise >  = 1 h everyday; Sufficient sleep: Sleep at least 8 h; Excessive screen time: Spending over 2 h time of screen; Mental Toughness Scale for Adolescents: The scale [18–72] is composed of six dimensions, including challenge [3–12], commitment [3–12], emotion control [3–12], life control [3–12], confidence in abilities [3–12], and interpersonal confidence [3–12], with a higher score indicating higher mental toughness. MTS > 46 was classified into High group.

R^2^ = 0.156; Adjusted R^2^ = 0.146.

Significant values are in bold.

The results of linear regression analyses between factors and health literacy subdomains are presented in Tables 3, 4, 5, 6, 7, 8, 9, 10**.** Adolescents of older age, those with higher SES, those with healthy dietary habits, and those who exhibited higher levels of mental toughness consistently predicted better scores across most subdomains. Conversely, male gender and engagement in risky behaviors were generally associated with poorer scores. Detailed information is provided as follows.

**Table 3:** Factors associated with the “Gain access to” section of the Health Literacy Measure for Adolescents among the participants.

Variable	Univariable beta* (95%CI)	p-value	Multivariable beta (95%CI)	p-value
Age group				
< 13 years	reference		reference	
13–14 years	− 1.35 (− 3.98, 1.28)	0.313	0.95 (− 1.58, 3.48)	0.461
> 14 years	− 1.29 (− 3.90, 1.33)	0.334	1.19 (− 1.36, 3.75)	0.360
Sex				
Female	reference		reference	
Male	− 1.99 (− 4.17, 0.20)	0.075	− 3.64 (− 5.86, − 1.43)	**0.001**
SES [0–9]				
Low [0–2]	reference		reference	
Mid [3–5]	3.28 (0.22, 6.34)	**0.036**	2.96 (0.02, 5.89)	**0.048**
High [6–9]	7.57 (4.35, 10.79)	** < 0.001**	5.95 (2.83, 9.08)	** < 0.001**
Smoking				
No	reference		reference	
Yes	− 8.65 (− 17.25, − 0.05)	**0.049**	− 4.14 (− 12.52, 4.23)	0.332
Alcohol drinking				
No	reference		reference	
Yes	− 6.48 (− 10.39, − 2.57)	**0.001**	− 5.15 (− 9.03, − 1.26)	**0.009**
*Having breakfast 7 days in a week*				
No	reference		reference	
Yes	3.85 (1.69, 6.01)	** < 0.001**	1.88 (− 0.27, 4.03)	0.086
*Sufficient vegetable intake*				
No	reference		reference	
Yes	5.67 (2.55, 8.79)	** < 0.001**	3.94 (0.89, 6.98)	**0.011**
*Sufficient fruit intake*				
No	reference		reference	
Yes	5.72 (2.78, 8.65)	** < 0.001**	3.06 (0.16, 5.96)	**0.038**
*Sufficient physical activity*				
No	reference		reference	
Yes	3.27 (0.04, 6.50)	**0.047**	1.60 (− 1.59, 4.79)	0.326
*Sufficient sleep*				
No	reference		reference	
Yes	0.93 (− 1.26, 3.11)	0.406	− 0.28 (− 2.44, 1.88)	0.797
*Excessive screen time on video*				
No	reference		reference	
Yes	0.27 (− 1.93, 2.47)	0.810	1.52 (− 0.71, 3.76)	0.181
*Excessive screen time on electronic game*				
No	reference		reference	
Yes	− 3.21 (− 5.38, − 1.05)	**0.004**	− 0.93 (− 3.18, 1.32)	0.416
*Excessive screen time on social media*				
No	reference		reference	
Yes	− 1.00 (− 3.17, 1.17)	0.366	− 0.07 (− 2.34, 2.20)	0.953
*Self-reported obesity*				
No	reference		reference	
Yes	− 1.33 (− 3.63, 0.96)	0.254	− 0.48 (− 2.68, 1.72)	0.670
*MTS-A*				
Low	reference		reference	
High	11.50 (9.40, 13.60)	** < 0.001**	11.04 (8.88, 13.20)	** < 0.001**

*In linear regression, the beta coefficient indicates the influence of an independent variable on the dependent variable. A one-unit increase in the independent variable results in an average change of the beta coefficient in the dependent variable. Positive values indicate a positive correlation, while negative values indicate a negative correlation.

CI: confidence interval; SES: Socio-economic status; Sufficient vegetable intake: Had at least 3 servings of vegetables everyday; Sufficient fruit intake: Had at least 2 servings of fruit everyday; Sufficient physical activity: Moderate/Vigorous exercise >  = 1 h everyday; Sufficient sleep: Sleep at least 8 h; Excessive screen time: Spending over 2 h time of screen; Mental Toughness Scale for Adolescents: The scale [18–72] is composed of six dimensions, including challenge [3–12], commitment [3–12], emotion control [3–12], life control [3–12], confidence in abilities [3–12], and interpersonal confidence [3–12], with a higher score indicating higher mental toughness. MTS > 46 was classified into High group.

R^2^ = 0.115; Adjusted R^2^ = 0.104.

Significant values are in bold.

**Table 4:** Factors associated with the “Numeracy” section of the Health Literacy Measure for Adolescents among the participants.

Variable	Univariable beta* (95%CI)	p-value	Multivariable beta (95%CI)	p-value
Age group				
< 13 years	reference		reference	
13–14 years	3.75 (− 0.37, 7.87)	0.075	4.53 (0.36, 8.70)	**0.033**
> 14 years	17.68 (13.59, 21.78)	** < 0.001**	18.59 (14.39, 22.79)	** < 0.001**
Sex				
Female	reference		reference	
Male	− 0.99 (− 4.51, 2.53)	0.580	− 1.75 (− 5.39, 1.90)	0.347
SES [0–9]				
Low [0–2]	reference		reference	
Mid [3–5]	1.93 (− 3.03, 6.89)	0.446	3.11 (− 1.72, 7.94)	0.207
High [6–9]	0.78 (− 4.44, 5.99)	0.771	2.79 (− 2.34, 7.93)	0.286
Smoking				
No	reference		reference	
Yes	− 11.44 (− 25.27, 2.39)	0.105	− 14.28 (− 28.05, − 0.50)	0.042
Alcohol drinking				
No	reference		reference	
Yes	− 0.06 (− 6.36, 6.25)	0.986	− 1.97 (− 8.35, 4.42)	0.546
*Having breakfast 7 days in a week*				
No	reference		reference	
Yes	− 1.27 (− 4.76, 2.22)	0.476	− 1.22 (− 4.76, 2.32)	0.500
*Sufficient vegetable intake*				
No	reference		reference	
Yes	− 4.89 (− 9.93, 0.15)	0.057	− 4.90 (− 9.91, 0.10)	0.055
*Sufficient fruit intake*				
No	reference		reference	
Yes	− 7.33 (− 12.06, − 2.60)	**0.002**	− 6.06 (− 10.83, − 1.29)	**0.013**
*Sufficient physical activity*				
No	reference		reference	
Yes	2.37 (− 2.83, 7.57)	0.372	4.69 (− 0.56, 9.94)	0.080
*Sufficient sleep*				
No	reference		reference	
Yes	− 2.46 (− 5.97, 1.05)	0.169	− 1.15 (− 4.70, 2.41)	0.527
*Excessive screen time on video*				
No	reference		reference	
Yes	0.21 (− 3.33, 3.75)	0.907	1.51 (− 2.16, 5.19)	0.419
*Excessive screen time on electronic game*				
No	reference		reference	
Yes	− 4.23 (− 7.72, − 0.75)	**0.017**	− 2.96 (− 6.65, 0.74)	0.117
*Excessive screen time on social media*				
No	reference		reference	
Yes	− 2.29 (− 5.78, 1.20)	0.199	− 3.64 (− 7.37, 0.10)	0.056
*Self-reported obesity*				
No	reference		reference	
Yes	2.19 (− 1.50, 5.88)	0.245	1.70 (− 1.91, 5.32)	0.356
*MTS-A*				
Low	reference		reference	
High	3.16 (− 0.35, 6.66)	0.078	4.27 (0.71, 7.82)	**0.019**

*In linear regression, the beta coefficient indicates the influence of an independent variable on the dependent variable. A one-unit increase in the independent variable results in an average change of the beta coefficient in the dependent variable. Positive values indicate a positive correlation, while negative values indicate a negative correlation.

CI: confidence interval; SES: Socio-economic status; Sufficient vegetable intake: Had at least 3 servings of vegetables everyday; Sufficient fruit intake: Had at least 2 servings of fruit everyday; Sufficient physical activity: Moderate/Vigorous exercise >  = 1 h everyday; Sufficient sleep: Sleep at least 8 h; Excessive screen time: Spending over 2 h time of screen; Mental Toughness Scale for Adolescents: The scale [18–72] is composed of six dimensions, including challenge [3–12], commitment [3–12], emotion control ^3–12^, life control [3–12], confidence in abilities [3–12], and interpersonal confidence [3–12], with a higher score indicating higher mental toughness. MTS > 46 was classified into High group.

R^2^ = 0.074; Adjusted R^2^ = 0.063.

Significant values are in bold.

**Table 5:** Factors associated with the “Communication” section of the Health Literacy Measure for Adolescents among the participants.

Variable	Univariable beta* (95%CI)	p-value	Multivariable beta (95%CI)	p-value
Age group				
< 13 years	reference		reference	
13–14 years	0.10 (− 2.53, 2.73)	0.940	2.67 (0.17, 5.17)	**0.036**
> 14 years	1.21 (− 1.40, 3.83)	0.362	3.99 (1.48, 6.51)	**0.002**
Sex				
Female	reference		reference	
Male	− 2.32 (− 4.51, − 0.14)	**0.037**	− 4.12 (− 6.31, − 1.94)	** < 0.001**
SES [0–9]				
Low [0–2]	reference		reference	
Mid ^3–5^	4.05 (0.99, 7.11)	**0.009**	3.73 (0.84, 6.62)	**0.012**
High ^6–9^	8.12 (4.91, 11.34)	** < 0.001**	6.48 (3.41, 9.56)	** < 0.001**
Smoking				
No	reference		reference	
Yes	− 10.70 (− 19.29, − 2.11)	**0.015**	− 6.88 (− 15.13, 1.38)	0.102
Alcohol drinking				
No	reference		reference	
Yes	− 5.44 (− 9.35, − 1.53)	**0.006**	− 3.93 (− 7.75, − 0.11)	**0.044**
*Having breakfast 7 days in a week*				
No	reference		reference	
Yes	2.02 (− 0.15, 4.19)	0.068	− 0.52 (− 2.64, 1.60)	0.629
*Sufficient vegetable intake*				
No	reference		reference	
Yes	4.09 (0.96, 7.22)	**0.010**	1.67 (− 1.33, 4.67)	0.275
*Sufficient fruit intake*				
No	reference		reference	
Yes	6.70 (3.77, 9.62)	** < 0.001**	4.68 (1.82, 7.53)	**0.001**
*Sufficient physical activity*				
No	reference		reference	
Yes	4.00 (0.77, 7.23)	**0.015**	2.43 (− 0.71, 5.58)	0.129
*Sufficient sleep*				
No	reference		reference	
Yes	1.08 (− 1.11, 3.26)	0.333	0.23 (− 1.89, 2.36)	0.829
*Excessive screen time on video*				
No	reference		reference	
Yes	− 0.80 (− 3.00, 1.40)	0.476	0.90 (− 1.30, 3.10)	0.424
*Excessive screen time on electronic game*				
No	reference		reference	
Yes	− 5.26 (− 7.42, − 3.11)	** < 0.001**	− 2.66 (− 4.88, − 0.45)	**0.018**
*Excessive screen time on social media*				
No	reference		reference	
Yes	− 1.51 (− 3.68, 0.66)	0.172	− 0.42 (− 2.66, 1.82)	0.714
*Self-reported obesity*				
No	reference		reference	
Yes	− 2.07 (− 4.37, 0.22)	0.076	− 1.31 (− 3.47, 0.86)	0.236
*MTS-A*				
Low	reference		reference	
High	12.62 (10.54, 14.71)	** < 0.001**	12.43 (10.30, 14.56)	** < 0.001**

*In linear regression, the beta coefficient indicates the influence of an independent variable on the dependent variable. A one-unit increase in the independent variable results in an average change of the beta coefficient in the dependent variable. Positive values indicate a positive correlation, while negative values indicate a negative correlation.

CI: confidence interval; SES: Socio-economic status; Sufficient vegetable intake: Had at least 3 servings of vegetables everyday; Sufficient fruit intake: Had at least 2 servings of fruit everyday; Sufficient physical activity: Moderate/Vigorous exercise >  = 1 h everyday; Sufficient sleep: Sleep at least 8 h; Excessive screen time: Spending over 2 h time of screen; Mental Toughness Scale for Adolescents: The scale [18–72] is composed of six dimensions, including challenge [3–12], commitment [3–12], emotion control [3–12], life control [3–12], confidence in abilities [3–12], and interpersonal confidence [3–12], with a higher score indicating higher mental toughness. MTS > 46 was classified into High group.

R^2^ = 0.140; Adjusted R^2^ = 0.130.

Significant values are in bold.

**Table 6:** Factors associated with the “Use” section of the Health Literacy Measure for Adolescents among the participants.

Variable	Univariable beta* (95%CI)	p-value	Multivariable beta (95%CI)	p-value
Age group				
< 13 years	reference		reference	
13–14 years	− 1.16 (− 3.95, 1.62)	0.412	1.27 (− 1.43, 3.96)	0.356
> 14 years	0.34 (− 2.43, 3.10)	0.812	2.69 (− 0.03, 5.40)	0.053
Sex				
Female	reference		reference	
Male	− 3.28 (− 5.59, − 0.97)	**0.005**	− 4.84 (− 7.20, − 2.49)	** < 0.001**
SES [0–9]				
Low [0–2]	reference		reference	
Mid ^3–5^	1.52 (− 1.73, 4.77)	0.360	0.93 (− 2.20, 4.05)	0.561
High ^6–9^	5.49 (2.07, 8.91)	**0.002**	3.35 (0.03, 6.67)	**0.048**
Smoking				
No	reference		reference	
Yes	− 9.17 (− 18.26, − 0.07)	**0.048**	− 6.37 (− 15.28, 2.53)	0.161
Alcohol drinking				
No	reference		reference	
Yes	− 3.76 (− 7.91, 0.38)	0.075	− 2.06 (− 6.19, 2.07)	0.328
*Having breakfast 7 days in a week*				
No	reference		reference	
Yes	2.87 (0.58, 5.17)	**0.014**	0.65 (− 1.64, 2.94)	0.578
*Sufficient vegetable intake*				
No	reference		reference	
Yes	7.13 (3.83, 10.42)	** < 0.001**	4.41 (1.17, 7.65)	**0.008**
*Sufficient fruit intake*				
No	reference		reference	
Yes	8.27 (5.18, 11.36)	** < 0.001**	5.88 (2.80, 8.97)	** < 0.001**
*Sufficient physical activity*				
No	reference		reference	
Yes	4.62 (1.21, 8.04)	**0.008**	3.11 (− 0.29, 6.50)	0.073
*Sufficient sleep*				
No	reference		reference	
Yes	1.95 (− 0.36, 4.26)	0.098	1.11 (− 1.18, 3.41)	0.342
*Excessive screen time on video*				
No	reference		reference	
Yes	− 2.86 (− 5.19, − 0.54)	**0.016**	− 1.63 (− 4.01, 0.75)	0.179
*Excessive screen time on electronic game*				
No	reference		reference	
Yes	− 5.15 (− 7.43, − 2.87)	** < 0.001**	− 2.21 (− 4.60, 0.19)	0.071
*Excessive screen time on social media*				
No	reference		reference	
Yes	− 0.72 (− 3.02, 1.58)	0.538	1.07 (− 1.35, 3.48)	0.386
*Self-reported obesity*				
No	reference		reference	
Yes	− 1.42 (− 3.85, 1.00)	0.250	− 0.66 (− 2.99, 1.68)	0.582
*MTS-A*				
Low	reference		reference	
High	10.53 (8.29, 12.77)	** < 0.001**	10.14 (7.85, 12.44)	** < 0.001**

*In linear regression, the beta coefficient indicates the influence of an independent variable on the dependent variable. A one-unit increase in the independent variable results in an average change of the beta coefficient in the dependent variable. Positive values indicate a positive correlation, while negative values indicate a negative correlation.

CI: confidence interval; SES: Socio-economic status; Sufficient vegetable intake: Had at least 3 servings of vegetables everyday; Sufficient fruit intake: Had at least 2 servings of fruit everyday; Sufficient physical activity: Moderate/Vigorous exercise >  = 1 h everyday; Sufficient sleep: Sleep at least 8 h; Excessive screen time: Spending over 2 h time of screen; Mental Toughness Scale for Adolescents: The scale [18–72] is composed of six dimensions, including challenge [3–12], commitment [3–12], emotion control [3–12], life control [3–12], confidence in abilities [3–12], and interpersonal confidence [3–12], with a higher score indicating higher mental toughness. MTS > 46 was classified into High group.

R^2^ = 0.106; Adjusted R^2^ = 0.095.

Significant values are in bold.

**Table 7:** Factors associated with the “Appraisal” section of the Health Literacy Measure for Adolescents among the participants.

Variable	Univariable beta* (95%CI)	p-value	Multivariable beta (95%CI)	p-value
Age group				
< 13 years	reference		reference	
13–14 years	− 0.53 (− 3.20, 2.15)	0.700	1.61 (− 0.97, 4.19)	0.221
> 14 years	1.21 (− 1.45, 3.86)	0.373	3.33 (0.73, 5.94)	**0.012**
Sex				
Female	reference		reference	
Male	− 2.35 (− 4.57, − 0.13)	**0.038**	− 3.72 (− 5.98, − 1.47)	**0.001**
SES [0–9]				
Low [0–2]	reference		reference	
Mid ^3–5^	2.53 (− 0.60, 5.65)	0.113	2.21 (− 0.78, 5.20)	0.148
High ^6–9^	5.60 (2.31, 8.88)	**0.001**	4.12 (0.94, 7.31)	**0.011**
Smoking				
No	reference		reference	
Yes	− 11.03 (− 19.76, − 2.30)	**0.013**	− 7.22 (− 15.76, 1.32)	0.097
Alcohol drinking				
No	reference		reference	
Yes	− 5.49 (− 9.46, − 1.51)	**0.007**	− 4.34 (− 8.30, − 0.39)	**0.031**
*Having breakfast 7 days in a week*				
No	reference		reference	
Yes	1.49 (− 0.72, 3.70)	0.185	− 0.42 (− 2.61, 1.78)	0.710
*Sufficient vegetable intake*				
No	reference		reference	
Yes	5.65 (2.47, 8.82)	** < 0.001**	3.85 (0.75, 6.95)	**0.015**
*Sufficient fruit intake*				
No	reference		reference	
Yes	4.87 (1.88, 7.85)	**0.001**	2.88 (− 0.08, 5.83)	0.056
*Sufficient physical activity*				
No	reference		reference	
Yes	2.51 (− 0.77, 5.80)	0.134	1.20 (− 2.05, 4.45)	0.469
*Sufficient sleep*				
No	reference		reference	
Yes	− 0.12 (− 2.34, 2.10)	0.918	− 0.80 (− 3.00, 1.40)	0.474
*Excessive screen time on video*				
No	reference		reference	
Yes	− 0.21 (− 2.45, 2.02)	0.853	1.12 (− 1.15, 3.40)	0.333
*Excessive screen time on electronic game*				
No	reference		reference	
Yes	− 4.19 (− 6.39, − 2.00)	** < 0.001**	− 2.03 (− 4.33, 0.26)	0.082
*Excessive screen time on social media*				
No	reference		reference	
Yes	− 0.96 (− 3.16, 1.25)	0.396	− 0.18 (− 2.50, 2.13)	0.876
*Self-reported obesity*				
No	reference		reference	
Yes	− 0.43 (− 2.77, 1.90)	0.715	0.19 (− 2.05, 2.43)	0.866
*MTS-A*				
Low	reference		reference	
High	11.67 (9.54, 13.80)	** < 0.001**	11.79 (9.59, 13.99)	** < 0.001**

*In linear regression, the beta coefficient indicates the influence of an independent variable on the dependent variable. A one-unit increase in the independent variable results in an average change of the beta coefficient in the dependent variable. Positive values indicate a positive correlation, while negative values indicate a negative correlation.

CI: confidence interval; SES: Socio-economic status; Sufficient vegetable intake: Had at least 3 servings of vegetables everyday; Sufficient fruit intake: Had at least 2 servings of fruit everyday; Sufficient physical activity: Moderate/Vigorous exercise >  = 1 h everyday; Sufficient sleep: Sleep at least 8 h; Excessive screen time: Spending over 2 h time of screen; Mental Toughness Scale for Adolescents: The scale [18–72] is composed of six dimensions, including challenge [3–12], commitment [3–12], emotion control [3–12], life control [3–12], confidence in abilities [3–12], and interpersonal confidence [3–12], with a higher score indicating higher mental toughness. MTS > 46 was classified into High group.

R^2^ = 0.110; Adjusted R^2^ = 0.099.

Significant values are in bold.

**Table 8:** Factors associated with the “Understanding” section of the Health Literacy Measure for Adolescents among the participants.

Variable	Univariable beta* (95%CI)	p-value	Multivariable beta (95%CI)	p-value
*Age group*				
< 13 years	reference		reference	
13–14 years	− 2.04 (− 4.60, 0.52)	0.118	0.37 (− 2.09, 2.84)	0.767
> 14 years	− 0.65 (− 3.20, 1.89)	0.614	1.70 (− 0.78, 4.19)	0.179
*Sex*				
Female	reference		reference	
Male	− 2.94 (− 5.07, − 0.81)	**0.007**	− 4.34 (− 6.50, − 2.18)	** < 0.001**
*SES [0–9]*				
Low [0–2]	reference		reference	
Mid ^3–5^	3.60 (0.60, 6.59)	**0.019**	3.30 (0.44, 6.16)	**0.024**
High ^6–9^	5.52 (2.37, 8.67)	**0.001**	3.96 (0.92, 7.00)	**0.011**
*Smoking*				
No	reference		reference	
Yes	− 13.31 (− 21.67, − 4.95)	**0.002**	− 8.08 (− 16.24, 0.08)	0.052
*Alcohol drinking*				
No	reference		reference	
Yes	− 7.40 (− 11.20, − 3.60)	** < 0.001**	− 5.47 (− 9.25, − 1.69)	**0.005**
*Having breakfast 7 days in a week*				
No	reference		reference	
Yes	3.79 (1.69, 5.90)	** < 0.001**	1.90 (− 0.19, 4.00)	0.075
*Sufficient vegetable intake*				
No	reference		reference	
Yes	4.54 (1.49, 7.58)	**0.004**	2.93 (− 0.04, 5.89)	0.053
*Sufficient fruit intake*				
No	reference		reference	
Yes	5.29 (2.43, 8.15)	** < 0.001**	3.06 (0.24, 5.89)	**0.034**
*Sufficient physical activity*				
No	reference		reference	
Yes	1.91 (− 1.24, 5.06)	0.235	0.99 (− 2.12, 4.10)	0.532
*Sufficient sleep*				
No	reference		reference	
Yes	− 0.07 (− 2.20, 2.06)	0.951	− 1.32 (− 3.42, 0.79)	0.219
*Excessive screen time on video*				
No	reference		reference	
Yes	− 0.18 (− 2.33, 1.96)	0.868	1.72 (− 0.45, 3.90)	0.121
*Excessive screen time on electronic game*				
No	reference		reference	
Yes	− 4.77 (− 6.87, − 2.67)	** < 0.001**	− 2.20 (− 4.39, − 0.01)	**0.049**
*Excessive screen time on social media*				
No	reference		reference	
Yes	− 2.60 (− 4.71, − 0.49)	**0.016**	− 1.65 (− 3.86, 0.57)	0.145
*Self-reported obesity*				
No	reference		reference	
Yes	− 0.46 (− 2.70, 1.78)	0.687	0.39 (− 1.75, 2.54)	0.718
*MTS-A*				
Low	reference		reference	
High	10.79 (8.73, 12.84)	** < 0.001**	10.47 (8.36, 12.57)	** < 0.001**

*In linear regression, the beta coefficient indicates the influence of an independent variable on the dependent variable. A one-unit increase in the independent variable results in an average change of the beta coefficient in the dependent variable. Positive values indicate a positive correlation, while negative values indicate a negative correlation.

CI: confidence interval; SES: Socio-economic status; Sufficient vegetable intake: Had at least 3 servings of vegetables everyday; Sufficient fruit intake: Had at least 2 servings of fruit everyday; Sufficient physical activity: Moderate/Vigorous exercise >  = 1 h everyday; Sufficient sleep: Sleep at least 8 h; Excessive screen time: Spending over 2 h time of screen; Mental Toughness Scale for Adolescents: The scale [18–72] is composed of six dimensions, including challenge [3–12], commitment [3–12], emotion control [3–12], life control [3–12], confidence in abilities [3–12], and interpersonal confidence [3–12], with a higher score indicating higher mental toughness. MTS > 46 was classified into High group.

R^2^ = 0.115; Adjusted R^2^ = 0.105.

Significant values are in bold.

**Table 9:** Factors associated with the “Reading” section of the Health Literacy Measure for Adolescents among the participants.

Variable	Univariable beta* (95%CI)	p-value	Multivariable beta (95%CI)	p-value
*Age group*				
< 13 years	reference		reference	
13–14 years	− 1.44 (− 4.39, 1.51)	0.338	0.94 (− 1.93, 3.82)	0.520
> 14 years	0.86 (− 2.08, 3.79)	0.567	3.22 (0.32, 6.12)	**0.030**
*Sex*				
Female	reference		reference	
Male	− 3.64 (− 6.09, − 1.19)	**0.004**	− 5.02 (− 7.54, − 2.50)	** < 0.001**
*SES [0–9*]				
Low [0–2]	reference		reference	
Mid ^3–5^	2.87 (− 0.58, 6.32)	0.103	2.52 (− 0.82, 5.85)	0.139
High ^6–9^	6.10 (2.47, 9.73)	**0.001**	4.38 (0.83, 7.92)	**0.016**
*Smoking*				
No	reference		reference	
Yes	− 12.89 (− 22.53, − 3.24)	**0.009**	− 9.09 (− 18.60, 0.43)	0.061
*Alcohol drinking*				
No	reference		reference	
Yes	− 5.05 (− 9.44, − 0.65)	**0.024**	− 3.16 (− 7.57, 1.24)	0.159
*Having breakfast 7 days in a week*				
No	reference		reference	
Yes	3.34 (0.91, 5.77)	**0.007**	1.44 (− 1.00, 3.89)	0.247
*Sufficient vegetable intake*				
No	reference		reference	
Yes	5.58 (2.07, 9.09)	**0.002**	3.47 (0.01, 6.93)	**0.049**
*Sufficient fruit intake*				
No	reference		reference	
Yes	6.32 (3.03, 9.62)	** < 0.001**	4.17 (0.88, 7.47)	**0.013**
*Sufficient physical activity*				
No	reference		reference	
Yes	0.98 (− 2.65, 4.61)	0.596	− 0.20 (− 3.82, 3.43)	0.916
*Sufficient sleep*				
No	reference		reference	
Yes	0.89 (− 1.56, 3.34)	0.476	0.13 (− 2.32, 2.59)	0.916
*Excessive screen time on video*				
No	reference		reference	
Yes	− 1.68 (− 4.15, 0.79)	0.182	− 0.47 (− 3.01, 2.07)	0.715
*Excessive screen time on electronic game*				
No	reference		reference	
Yes	− 4.22 (− 6.65, − 1.79)	**0.001**	− 1.34 (− 3.90, 1.21)	0.302
*Excessive screen time on social media*				
No	reference		reference	
Yes	− 1.16 (− 3.60, 1.28)	0.350	0.21 (− 2.37, 2.79)	0.874
*Self-reported obesity*				
No	reference		reference	
Yes	− 0.44 (− 3.02, 2.14)	0.738	0.27 (− 2.22, 2.77)	0.830
*MTS-A*				
Low	reference		reference	
High	11.24 (8.86, 13.62)	** < 0.001**	11.31 (8.86, 13.77)	** < 0.001**

*In linear regression, the beta coefficient indicates the influence of an independent variable on the dependent variable. A one-unit increase in the independent variable results in an average change of the beta coefficient in the dependent variable. Positive values indicate a positive correlation, while negative values indicate a negative correlation.

CI: confidence interval; SES: Socio-economic status; Sufficient vegetable intake: Had at least 3 servings of vegetables everyday; Sufficient fruit intake: Had at least 2 servings of fruit everyday; Sufficient physical activity: Moderate/Vigorous exercise >  = 1 h everyday; Sufficient sleep: Sleep at least 8 h; Excessive screen time: Spending over 2 h time of screen; Mental Toughness Scale for Adolescents: The scale [18–72] is composed of six dimensions, including challenge [3–12], commitment [3–12], emotion control [3–12], life control [3–12], confidence in abilities [3–12], and interpersonal confidence [3–12], with a higher score indicating higher mental toughness. MTS > 46 was classified into High group.

R^2^ = 0.094; Adjusted R^2^ = 0.083.

Significant values are in bold.

**Table 10:** Factors associated with the “Self-efficacy” section of the Health Literacy Measure for Adolescents among the participants.

Variable	Univariable beta* (95%CI)	p-value	Multivariable beta (95%CI)	p-value
*Age group*				
< 13 years	reference		reference	
13–14 years	− 0.54 (− 3.10, 2.03)	0.682	1.59 (− 0.89, 4.07)	0.208
> 14 years	0.66 (− 1.89, 3.21)	0.613	3.12 (0.63, 5.62)	**0.014**
*Sex*				
Female	reference		reference	
Male	− 0.23 (− 2.37, 1.90)	0.831	− 1.68 (− 3.85, 0.49)	0.128
*SES [0–9]*				
Low [0–2]	reference		reference	
Mid ^3–5^	3.49 (0.51, 6.48)	**0.022**	3.40 (0.53, 6.27)	**0.020**
High ^6–9^	7.69 (4.55, 10.83)	** < 0.001**	6.44 (3.39, 9.49)	** < 0.001**
*Smoking*				
No	reference		reference	
Yes	− 8.13 (− 16.53, 0.26)	0.058	− 4.59 (− 12.78, 3.60)	0.272
*Alcohol drinking*				
No	reference		reference	
Yes	− 4.99 (− 8.81, − 1.17)	**0.011**	− 3.45 (− 7.24, 0.35)	0.075
*Having breakfast 7 days in a week*				
No	reference		reference	
Yes	4.59 (2.49, 6.70)	** < 0.001**	2.39 (0.29, 4.49)	**0.026**
*Sufficient vegetable intake*				
No	reference		reference	
Yes	4.24 (1.19, 7.30)	**0.007**	2.43 (− 0.55, 5.41)	0.109
*Sufficient fruit intake*				
No	reference		reference	
Yes	5.88 (3.02, 8.75)	** < 0.001**	3.48 (0.64, 6.31)	**0.016**
*Sufficient physical activity*				
No	reference		reference	
Yes	2.51 (− 0.64, 5.67)	0.118	0.28 (− 2.84, 3.40)	0.860
*Sufficient sleep*				
No	reference		reference	
Yes	2.14 (0.01, 4.27)	**0.049**	0.90 (− 1.21, 3.02)	0.401
*Excessive screen time on video*				
No	reference		reference	
Yes	− 0.99 (− 3.14, 1.16)	0.365	0.46 (− 1.73, 2.64)	0.680
*Excessive screen time on electronic game*				
No	reference		reference	
Yes	− 4.19 (− 6.30, − 2.08)	** < 0.001**	− 1.99 (− 4.19, 0.21)	0.076
*Excessive screen time on social media*				
No	reference		reference	
Yes	− 1.54 (− 3.66, 0.58)	0.155	0.22 (− 2.00, 2.44)	0.843
*Self-reported obesity*				
No	reference		reference	
Yes	− 1.17 (− 3.42, 1.07)	0.304	− 0.24 (− 2.39, 1.91)	0.826
*MTS-A*				
Low	reference		reference	
High	11.28 (9.23, 13.33)	** < 0.001**	10.51 (8.40, 12.63)	** < 0.001**

*In linear regression, the beta coefficient indicates the influence of an independent variable on the dependent variable. A one-unit increase in the independent variable results in an average change of the beta coefficient in the dependent variable. Positive values indicate a positive correlation, while negative values indicate a negative correlation.

CI: confidence interval; SES: Socio-economic status; Sufficient vegetable intake: Had at least 3 servings of vegetables everyday; Sufficient fruit intake: Had at least 2 servings of fruit everyday; Sufficient physical activity: Moderate/Vigorous exercise >  = 1 h everyday; Sufficient sleep: Sleep at least 8 h; Excessive screen time: Spending over 2 h time of screen; Mental Toughness Scale for Adolescents: The scale [18–72] is composed of six dimensions, including challenge [3–12], commitment [3–12], emotion control [3–12], life control [3–12], confidence in abilities [3–12], and interpersonal confidence [3–12], with a higher score indicating higher mental toughness. MTS > 46 was classified into High group.

R^2^ = 0.113; Adjusted R^2^ = 0.102.

Significant values are in bold.

Table 3 presents factors related to the ability to access health information. Alcohol use (β =  − 5.15, 95% CI: − 9.03 to − 1.26, *p* = 0.009) and male gender (β =  − 3.64, 95% CI: − 5.86 to − 1.43, *p* = 0.001) were linked to poorer access. In contrast, higher SES (β_medium = 2.96, 95% CI: 0.02–5.89, *p* = 0.048; β_high = 5.95, 95% CI: 2.83–9.08, *p* < 0.001), adequate intake of vegetables (β = 3.94, 95% CI: 0.89–6.98, *p* = 0.011) and fruits (β = 3.06, 95% CI: 0.16–5.96, *p* = 0.038), and stronger mental toughness (β = 11.04, 95% CI: 8.88–13.02, *p* < 0.001) were associated with enhanced information access.

Regarding numeracy in health (Table 4), older adolescents (β_13–14 = 4.53, 95% CI: 0.36–8.70, *p* = 0.033; β_ > 14 = 18.59, 95% CI: 14.39–22.79, *p* < 0.001) and those with greater mental toughness (β = 4.27, 95% CI: 0.71–7.82, *p* = 0.019) exhibited stronger performance. Surprisingly, an inverse association was observed, as sufficient fruit consumption was inversely related to numeracy ability (β =  − 6.06, 95% CI: − 10.83 to − 1.29, *p* = 0.013), which warrants further investigation.

Table 5 shows that better communication of health information was associated with older age (β_13–14 = 2.67, 95% CI: 0.17–5.17, *p* = 0.036; β_ > 14 = 3.99, 95% CI: 1.48–6.51, *p* = 0.002), higher SES (β_medium = 3.73, 95% CI: 0.84–6.62, *p* = 0.012; β_high = 6.48, 95% CI: 3.41–9.56, *p* < 0.001), and mental toughness (β = 12.43, 95% CI: 10.30–14.56, *p* < 0.001). In contrast, males (β =  − 4.12, 95% CI: − 6.31 to − 1.94, *p* < 0.001), those who consumed alcohol (β =  − 3.93, 95% CI: − 7.75 to − 0.11, *p* = 0.044), and had excessive screen time on electronic game(β =  − 2.66, 95% CI: − 4.88 to − 0.45, *p* = 0.018) had lower communication scores.

As illustrated in Table 6, adolescents with high SES (β = 3.35, 95% CI: 0.03–6.67, *p* = 0.048), sufficient vegetable (β = 4.41, 95% CI: 1.17–7.65, *p* = 0.008) and fruit intake (β = 5.88, 95% CI: 2.80–8.97, *p* < 0.001), and high mental toughness (β = 10.14, 95% CI: 7.85–12.44, *p* < 0.001) were more adept at utilizing health information. Male adolescents lagged behind (β =  − 4.84, 95% CI: − 7.20 to − 2.49, *p* < 0.001).

Table 7 identifies predictors of health information appraisal. Males (β =  − 3.72, 95% CI: − 5.98 to − 1.47, *p* = 0.001) and alcohol users (β =  − 4.34, 95% CI: − 8.30 to − 0.39, *p* = 0.031) showed lower appraisal capacity. Meanwhile, those aged over 14 years (β = 3.33, 95% CI: 0.73–5.94, *p* = 0.012), with high SES (β = 4.12, 95% CI: 0.94–7.31, *p* = 0.011), adequate vegetable consumption (β = 3.85, 95% CI: 0.75–6.95, *p* = 0.015), and higher mental toughness (β = 11.79, 95% CI: 9.59–13.99, *p* < 0.001) showed better appraisal ability.

Table 8 presents factors influencing the ability to understand health information. Adolescents with higher SES (β_medium = 3.30, 95% CI: 0.44–6.16, *p* = 0.024; β_high = 3.96, 95% CI: 0.92–7.00, *p* = 0.011), fruit intake (β = 3.06, 95% CI: 0.24–5.89, *p* = 0.034), and stronger mental toughness (β = 10.47, 95% CI: 8.36–12.57, *p* < 0.001) performed better, while alcohol consumption (β =  − 5.47, 95% CI: − 9.25 to − 1.69, *p* = 0.005), excessive screen time on electronic game (β =  − 2.20, 95% CI: − 4.39 to − 0.01, *p* = 0.049), and being male (β =  − 4.34, 95% CI: − 6.50 to − 2.18, *p* < 0.001) were linked to poorer understanding.

As indicated in Table 9, male gender (β =  − 5.02, 95% CI: − 7.54 to − 2.50, *p* < 0.001) was the only factor negatively associated with reading ability. On the other hand, age over 14 (β = 3.22, 95% CI: 0.32–6.12, *p* = 0.030), high SES (β = 4.38, 95% CI: 0.83–7.92, *p* = 0.016), sufficient vegetable (β = 3.47, 95% CI: 0.01–6.93, *p* = 0.049) and fruit intake (β = 4.17, 95% CI: 0.88–7.47, *p* = 0.013), and greater mental toughness (β = 11.31, 95% CI: 8.86–13.77, *p* < 0.001) all favored improved reading ability.

Last but not least, Table 10 summarizes the correlates of self-efficacy. Higher self-efficacy scores were observed among older adolescents (β = 3.12, 95% CI: 0.63–5.62, *p* = 0.014), those with higher SES (β_medium = 3.40, 95% CI: 0.53–6.27, *p* = 0.020; β_high = 6.44, 95% CI: 3.39–9.49, *p* < 0.001), those with regular breakfast habits (β = 2.39, 95% CI: 0.29–4.49, *p* = 0.026), adequate fruit intake (β = 3.48, 95% CI: 0.64–6.31, *p* = 0.016), and those with higher mental toughness (β = 10.51, 95% CI: 8.40–12.63, *p* < 0.001).

## Discussion

### Major findings

This study comprehensively investigated the factors associated with health literacy among adolescents in Hong Kong. The following key findings were identified: (1) The level of health literacy among the adolescents surveyed was alarming, especially in terms of self-efficacy. (2) Adolescent health literacy was associated with higher SES, older age, healthy behaviors, higher levels of mental toughness, and less screen time for electronic games. (3) Mental toughness was associated with all health literacy sub-domain and demonstrated strong effects.

### Evidence from previous research

Our study found that the health literacy of Hong Kong secondary students was poor, with an average score of 57.24 (SD = 17.04). Similarly, a previous survey of students across 10 Hong Kong secondary schools revealed notably low health literacy levels among adolescents, with approximately 74.4% demonstrating limited health literacy^15^. This appears to be a widespread phenomenon, not unique to Hong Kong but prevalent globally. For example, a scoping review of 82 studies reported that more than half of the studies found low or moderate health literacy levels among over 50% of adolescents and young adults^39^. Although only 22.7% of German adolescents aged 14–17 had a low level of health knowledge, majority (50.58%) at moderate levels^40^. Furthermore, self-efficacy was found to be particularly problematic among Hong Kong adolescents. The previous Hong Kong survey also found self-efficacy as one of the lowest scores reported by students^15^, which underscores the importance of identifying at-risk groups early on to enable timely intervention.

To explain the observed higher prevalence of low health literacy among Hong Kong adolescents, our study identified several factors associated with health literacy. By sub-domain analysis, we found strong positive effects of mental toughness on all sub-domains of health literacy. Mental toughness is regarded as a resilience resource, typically referring to an individual’s adaptability in growth and pursuing development opportunities, particularly in the face of adversity^37^. Previous research indicates that adolescents with high levels of resilience resources are more likely to demonstrate positive health behaviors than those with lower resilience, including increased physical activity, healthier dietary habits, and maintaining a healthy body weight^41^. This observation may be attributed to an increased availability of resilience resources, which promote more positive psychological functioning, thereby facilitating individuals’ efforts to attain improved physiological health through the enhancement of their personal capabilities^42^.

This study found adolescents with medium or high SES had higher health literacy than those with low SES. SES is associated with different health related lifestyles and health outcomes, known as one of the most fundamental causes of health disparity^43^ Many studies have identified health literacy as a significant mediator between socioeconomic status (SES) and health-related outcomes^44–47^. A previous Italian study of 452 subjects reported that functional health literacy mediated 18.5% of the association between education and self-rated health, and 12.9% of the relationship between financial status and self-reported health^46^. The impact of SES on the health literacy of adolescents was also explored by a cross-sectional study of Brazilian adolescents, which found oral health literacy to be significantly associated with both high mother’s schooling level and high income^48^. Families with higher SES possess greater resources to invest in their children’s education, thereby enhancing their learning capacity and ability to comprehend, analyze, and utilize health-related information.

Healthy dietary behaviors, such as adequate vegetable intake and fruit consumption, have been found to correlated with higher health literacy. This phenomenon may be attributed to the influence exerted by the familial environment of adolescents and parental behavior. Due to the distribution of authority within families and the allocation of financial resources, parents are often deemed to play a pivotal role in shaping the dietary behaviors of adolescents and children^49–51^. A qualitative systematic review indicates that parental dietary preferences, knowledge, and food availability constitute key determinants of adolescents’ healthy eating habits^52^. Adolescents from low SES families are more likely to develop unhealthy eating habits compared to their counterparts from higher SES households or those with greater educational attainment^52^, who possess increased access to health-related knowledge, education, and health behavior regulation^53–55^.

In addition, risky behaviors, such as smoking and alcohol drinking, were negatively associated with healthy literacy, which has been identified by previous studies^56–59^. Although the specific reasons remain unclear, this may be attributed to the confounding effects of personal experience and growing environment among adolescents engaging in risky behaviors. For instance, the development of risky behavior, such as smoking, is typically associated with lower SES^60^, where individuals have a lower ability to access education and resources that promote healthy behaviors^61^, thereby constraining the development of their health literacy.

This study investigates the adverse effects of excessive screen time on health literacy, with particular emphasis on the duration spent on electronic games. However, mixed findings on the association between screen time and health literacy were reported by previous studies. A Chinese cross-sectional survey study found that while internet addiction was negatively associated with critical health literacy, and positively associated with functional and interactive health literacy, it was not significantly associated with overall health literacy^62^. On the contrary, another study from China emphasized that screen time of less than two hours is positively associated with nutritional literacy, potentially attributable to reduced exposure to information about unhealthy products and behaviors^63^. Likewise, previous studies in Hong Kong provided similar findings, indicating that more screen time spent on TV was associated with lower health literacy levels among the child population in Hong Kong^64^. The discrepancies observed between our findings and prior research may be attributed to variations in information exposure resulting from differing patterns of screen time among adolescents in diverse settings. For example, adolescents who are more frequently exposed to marketing that endorses unhealthy lifestyles may be more vulnerable to adverse influences^65^. Conversely, those participating in screen time activities, such as gaming that includes health-related components, are more likely to demonstrate increased health literacy^66,67^.

Gender disparity in health literacy was significant, with boys having a lower health literacy than girls. A comparative study of adolescents in 10 European countries, however, only identified three countries where had significant gender disparity in health literacy levels (with girls having higher health literacy) among adolescents, including Estonia, Macedonia, and Poland^68^. Another study found that females with cystic fibrosis had lower health literacy than their male counterparts, whereas an opposite finding was found in the general population^69^. This disparity may be attributed to male norms and the development of masculinity among adolescent males. Prior research indicates that certain male norms, such as self-reliance and the impression of rationality, may limit males’ ability to actively seek health services or information and communicate about health issues^70–72^.

### Strengths and limitations

Our study had several strengths. Firstly, previously validated instruments were employed to assess health literacy, as well as psychological and behavioral factors, thereby improving the robustness of our measures. For instance, to measure the mental toughness of adolescents, we used the MTS-A, which has previously been widely used in prior research to examine its associations with health literacy^73–75^. The scales employed in this study demonstrated satisfactory performance, with moderate to high internal reliability from 0.34 to 0.96 for different scales. Secondly, our comprehensive selection of independent variables—including sociodemographic characteristics, health-related behaviors, and psychological attributes—allowed for a more robust exploration of potential determinants of health literacy, while minimizing confounding. Thirdly, the study benefits from a large sample size, which strengthens the statistical power and reliability of the findings.

However, there are also potential limitations. Most notably, the cross-sectional study design precludes any inference of causality. Longitudinal studies are warranted to explore the temporal and potential causal relationships between the identified factors and health literacy outcomes. In addition, the use of voluntary participation by schools and students may have introduced selection bias, as individuals who chose to participate might differ systematically from those who declined. This could potentially limit the representativeness of the sample and may lead to an overestimation of health literacy levels, considering that students with higher health literacy are more likely to participate in this study. Moreover, the design of self-administered questionnaires may introduce self-reporting bias, thereby affecting the accuracy of reported data. However, we provided visual materials and examples to assist participants in reporting data more accurately. The generalizability of the findings may also be constrained by the study’s geographical and cultural context; since the sample consisted solely of students from Hong Kong, the results may not be applicable to other populations or educational systems. Future research involving more diverse and cross-cultural samples is recommended to enhance the external validity of our findings. Furthermore, the limitation arising from the absence of a pilot test may marginally impact the refinement of the instrument and the robustness of the data collection process.

### Implications

Our study comprehensively examined factors associated with health literacy among Hong Kong adolescents and identified potential high-risk groups that may benefit from targeted interventions. The findings offer valuable insights for parents, schools, and policymakers in developing strategies to promote health literacy. For example, parents and educators can refer to the factors identified in this study to assess the health literacy and health behaviors of adolescents with similar characteristics, enabling early intervention. In addition, the demographic disparities highlighted in this study suggest that future school-based and community-level initiatives should be tailored to the specific needs of vulnerable subgroups and at-risk populations. For example, more targeted school-based health education programs tailored to the interests and preferences of adolescent males can be developed to enhance their participation. Policymakers may also use these findings to inform resource allocation and design targeted health promotion programs. Future research may consider incorporating high-risk groups identified into the design and evaluation of effective health literacy interventions for adolescents and validating their efficacy. Furthermore, our findings emphasize the necessity for longitudinal studies to investigate the causal relationships between the identified factors and health literacy, particularly concerning mental health toughness.

## Conclusion

In conclusion, this study identifies high-risk groups for health literacy among adolescents in Hong Kong, including males, those with lower SES, poor dietary habits, lower levels of mental toughness, engagement in risky behaviors such as smoking and alcohol drinking, and younger adolescents. Future research and policymakers can develop targeted interventions for these identified groups to optimize the effectiveness.

## Supplementary Information

Below is the link to the electronic supplementary material.

Supplementary Material 1

## Data Availability

The datasets used and/or analysed during the current study are available from the corresponding author on reasonable request.
