# Supplementary material for: Exploring the factors associated with health literacy among adolescents in Hong Kong
Source: Sci Rep. 2026 Jan 30;16:4320. doi: 10.1038/s41598-025-28900-y (PMC12864948; doi:10.1038/s41598-025-28900-y)

Supplementary Table 1. Variance Inflation Factors

|  | **GVIF** | **Df** | **GVIF^(1/(2*Df))** |
| --- | --- | --- | --- |
| Age group | 1.087675 | 2 | 1.021233 |
| Sex | 1.145249 | 1 | 1.070163 |
| Self-reported Obesity | 1.023759 | 1 | 1.01181 |
| Socio-economic Status | 1.044693 | 2 | 1.010991 |
| Daily Breakfast | 1.095848 | 1 | 1.046827 |
| Vegetable Intake | 1.051825 | 1 | 1.025585 |
| Fruit Intake | 1.079508 | 1 | 1.038994 |
| Physical Activity | 1.08779 | 1 | 1.042972 |
| Sleep Duration | 1.091237 | 1 | 1.044623 |
| Screen Time on Video | 1.151307 | 1 | 1.07299 |
| Screen Time on Electronic Game | 1.195293 | 1 | 1.093295 |
| Screen Time on Social Media | 1.221684 | 1 | 1.105298 |
| Smoking | 1.057683 | 1 | 1.028437 |
| Alcohol Drinking | 1.094147 | 1 | 1.046015 |
| Mental Toughness Level | 1.094295 | 1 | 1.046086 |

GVIF: Generalized Variance Inflation Factor; DF: Degree of Freedom

Supplementary Table 2. Intercorrelations Matrix

|  | **Gain access** | **Numeracy** | **Communication** | **Use** | **Appraisal** | **Understanding** | **Reading** | **Self-efficacy** |
| --- | --- | --- | --- | --- | --- | --- | --- | --- |
| Gain Access | 1.00 | 0.08 | 0.63 | 0.54 | 0.61 | 0.64 | 0.65 | 0.73 |
| Numeracy | 0.08 | 1.00 | 0.12 | 0.07 | 0.15 | 0.15 | 0.10 | 0.10 |
| Communication | 0.63 | 0.12 | 1.00 | 0.68 | 0.74 | 0.68 | 0.67 | 0.60 |
| Use | 0.54 | 0.07 | 0.68 | 1.00 | 0.65 | 0.53 | 0.52 | 0.50 |
| Appraisal | 0.61 | 0.15 | 0.74 | 0.65 | 1.00 | 0.80 | 0.69 | 0.54 |
| Understanding | 0.64 | 0.15 | 0.68 | 0.53 | 0.80 | 1.00 | 0.75 | 0.55 |
| Reading | 0.65 | 0.10 | 0.67 | 0.52 | 0.69 | 0.75 | 1.00 | 0.59 |
| Self-efficacy | 0.73 | 0.10 | 0.60 | 0.50 | 0.54 | 0.55 | 0.59 | 1.00 |

Supplementary Figure 1. Heatmap for Intercorrelation Matrix Among Health Literacy Subdomains


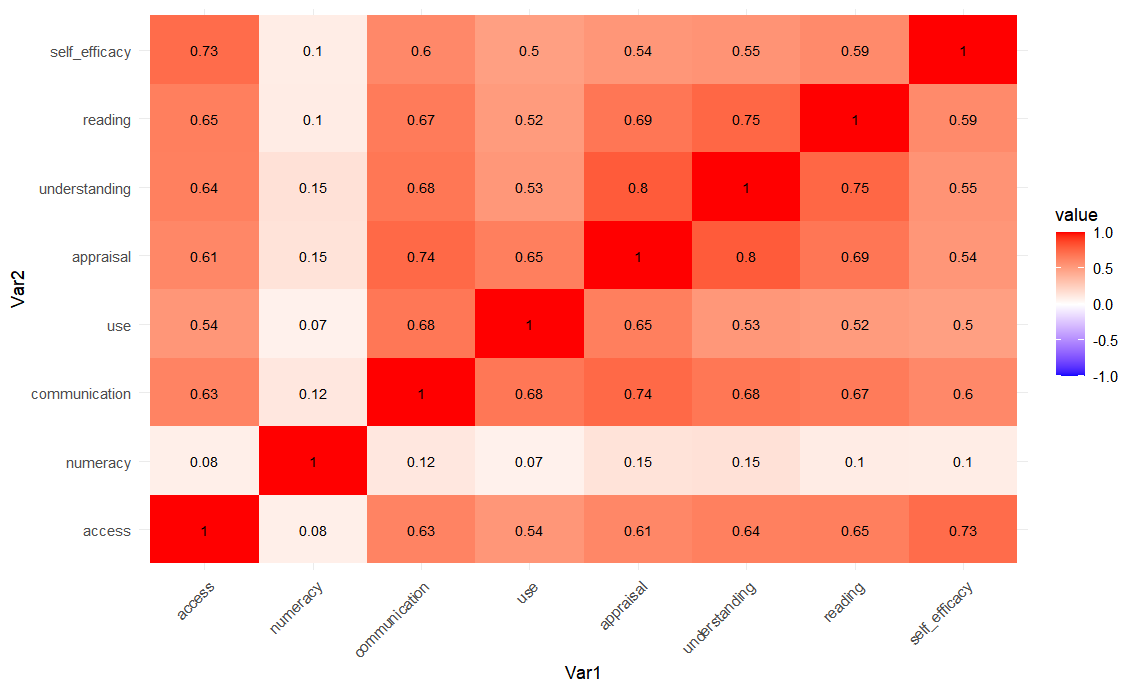

Supplement: Supplementary file 1 — Supplementary Material 1 [file 41598_2025_28900_MOESM1_ESM.docx]
